# Tottenham Hospital

**Published:** 1905-06-24

**Authors:** 


### TOTTENHAM HOSPITAL.

### GREAT EXTENSION CEREMONY.

The Lord Mayor, accompanied by the Lady Mayoress, the Master and several members of the Drapers' Company, assembled last Tuesday in the grounds of the Hospital for the purpose of laying the foundation-stones of the hospital extension. The proceedings consisted of two ceremonies, as the Lord Mayor laid the foundation-stone of one part of the extension, and the Master of the Drapers' Company that portion to be known as the "Drapers' Block." Sir Francis Cory Wright, chairman of the hospital, gave thanks to the Lord Mayor for his services, and referred to his activity in the cause of charity, and he expressed his gratitude to the Drapers' Company for their generosity towards the institution, which, amounting to £6,000, enabled the hospital to secure two new wards and an operating theatre. The proceedings terminated by an inspection of the hospital by the company present.

The Alterations and Additions to the Hospital.

The present nurses' dining-room is to be demolished, a new wing will be built on the site, which will contain wards on ground and first floors, and an operating theatre on the second floor with anæsthetising and surgeon's room adjoining, recovery and sterilising rooms. The theatre is to be top-lighted, its floor will be of marble mosaic, the walls lined with opalite, and it will have a narrow gallery above the floor level for the use of the staff. The wards will in every way be brought up to modern medical requirements, and each ward has a balcony for convalescent patients. The wards' sanitary annexés are in every case cut off by bridges, thus ensuring ample ventilation. Two of the present wards will be enlarged and modernised and a new residents' block will be erected. The entire existing drainage will also be taken up and relaid, and the whole of the heating apparatus—including hot and cold water—is being reconstructed; £20,000 is the sum stimated as required to complete the work.

